# The National Formulary of Unofficinal Preparations

**Published:** 1888-09

**Authors:** 


**The National Formulary of Unofficinal Preparations.** First issue. By authority of the American Pharmaceutical Association. 1888. 8vo. pp., IX. 176.

This little volume supplies the physician with the formulæ of many standard, yet unofficinal, preparations of an important nature. The editing committee of the American Pharmaceutical Association, under whose direction the work has been prepared, has performed its labors with great care, and the book should have a ready sale.

